# Supplementary material for: TCA cycle tailoring facilitates optimal growth of proton-pumping NADH dehydrogenase-dependent Escherichia coli
Source: Microbiol Spectr. 2023 Oct 19;11(6):e02225-23. doi: 10.1128/spectrum.02225-23 (PMC10715208; doi:10.1128/spectrum.02225-23)
Supplement: Supplemental figure, tables, and methods — Supplemental material. [file spectrum.02225-23-s0001.pdf]

SUPPLEMENTARY MATERIAL

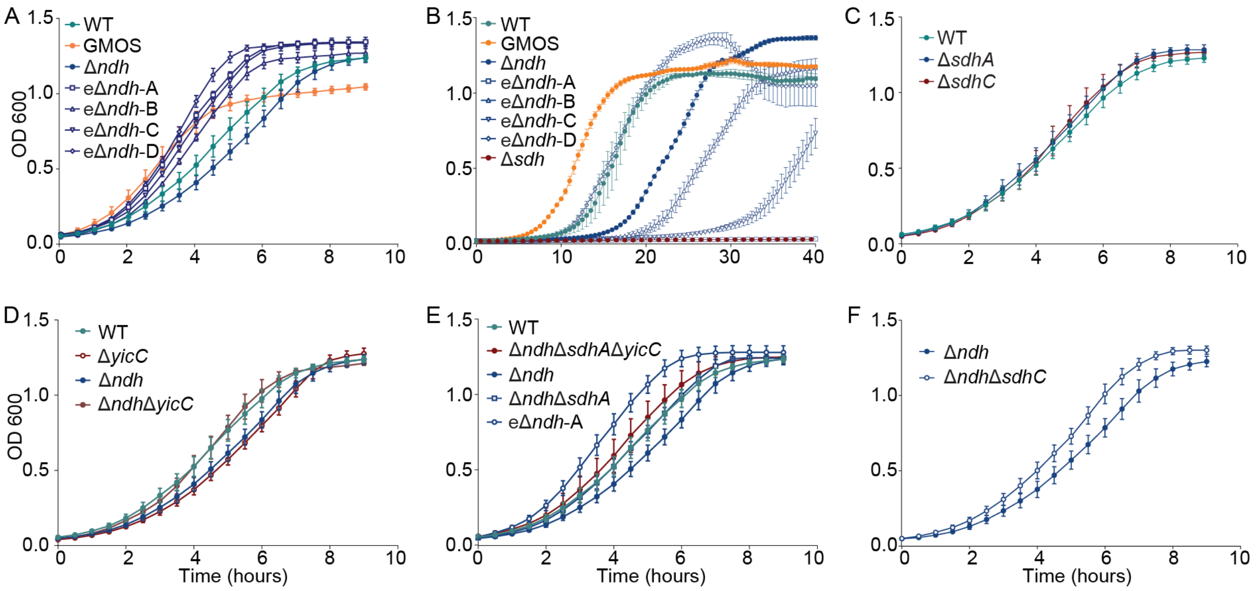

Supplementary Figure 1: (A) Growth comparison of unevolved and evolved strains of wild type and Δ*ndh* in M9 minimal medium with glucose. (B) The growth profile of the unevolved and evolved Δ*ndh* strains in M9 minimal medium with succinate. Wild Type (WT), GMOS, and Δ*sdhA* are included as a reference for the growth profile of *E. coli* on succinate. Δ*sdhA* and eΔ*ndh*-A showed complete failure in the growth and to represent both data, time-separated alternate OD values are plotted (C) Growth profile of the WT, Δ*sdhA*, and Δ*sdhC* in M9 minimal medium with glucose. (D) Growth profile of *yicC* deletion in WT and Δ*ndh* in M9 minimal medium with glucose. (E) Growth curve of WT, Δ*ndh*, Δ*ndh*Δ*sdhA* and Δ*ndh*Δ*sdhA*Δ*yicC* on M9 minimal medium with glucose. (F) Growth comparison of unevolved Δ*ndh* and Δ*ndh*Δ*sdhC* in M9 minimal medium with glucose. The growth curves in panel (A), (C) and (D-F) represent a mean of five biological replicates (with three technical replicates each). The growth curves in panel (B) represent a mean of three biological replicates (with three technical replicates each). In every plot, the error bars show the standard error of mean.

Supplementary Tables:

Supplementary Table 1: Sample code used during running the ALE machine

**Strain**	**ALE code**
Δ*ndh*	Δ*ndh*_A0F0I1R1
eΔ*ndh*-A	Δ*ndh*_A13F47I1R1
eΔ*ndh*-B	Δ*ndh*_A14F47I1R1
eΔ*ndh*-C	Δ*ndh*_A15F46I1R1
eΔ*ndh*-D	Δ*ndh*_A16F47I1R1

Supplementary Table 2: Genetic changes in evolved strains

Strain	Mutations		
eΔ*ndh*-A	*sdhA*<br>+T at 451	*yicC*<br>Δ103bp	
eΔ*ndh*-B	*sdhA*<br>C89R	*pyrE/rph* intergenic<br>C→A (-55/+11)	*fecR*<br>Q121L
eΔ*ndh*-C	*sdhA*<br>A414P	*yicC*<br>Δ103bp	
eΔ*ndh*-D	*sucA*<br>R182S	*pyrE/rph* intergenic<br>T→G (-47/+19)	*nusA*<br>A257E

List of mutations in evolved lineages of Δ*ndh* compared to unevolved strain.

Supplementary Table 3: Phenotypic characterization of the strains of the study

**Strain**	**Growth rate (per hour)**	**Glucose uptake rate (mmol/gDCW/h)**	**Acetate secretion rate (mmol/gDCW/h)**	**Lactate secretion rate (mmol/gDCW/h)**
Δ*ndh*	0.48, 0.50	6.74, 6.84	5.30, 5.56	0.23, 0.44
eΔ*ndh*-A	0.88, 0.84	10.75, 10.45	8.29, 7.75	N.D
eΔ*ndh*-B	0.80, 0.81	10.49, 10.17	8.07, 7.81	N.D.
eΔ*ndh*-C	0.83, 0.77	10.38, 9.76	7.57, 7.48	N.D.
eΔ*ndh*-D	0.90, 0.89	10.87, 10.53	5.98, 5.32	N.D.

N.D. stands for not detected.

Supplementary Table 4: Primers used for strain verification

**Primer name**	**Oligonucleotide Sequence (5' → 3')**
k1	CAGTCATAGCCGAATAGCCT
k2	CGGTGCCCTGAATGAACTGC
ndh (UP)	TGTTTTTTGATCTCACCCGG
ndh (DP)	TCCTTCTTTGCAGTTATGCC
sdhA (UP)	GGTTTCTTCGCCTCTGCGTT
sdhA (DP)	GAACCGCACACACCTTCACG
sdhC (UP)	TCCCGAGCCACCCAGCGTTG
sdhC (DP)	CAAGATAGAAAACAGCGCCA
yicC (UP)	GGGACGCACCTGATTATTGC
yicC (DP)	ACAGTTGTGAGTATCTCACG

UP: Upstream Primer; DP: Downstream Primer

Material and methods

Bacterial strains, media, and growth conditions

E. coli K-12 MG1655 (ATCC 700926) was used as the wild-type strain. The P1 phage transduction method was used to generate the knockout strains (1), and strains from the Keio collection were used as a donor for the gene knockout cassettes (2). Knockouts were confirmed by PCR (primers are listed in Supplementary Table 4). M9 minimal medium supplemented with 4 g/L glucose was used for bacterial growth unless stated otherwise.

Growth characterizations

Strains were grown at 37°C using an orbital shaker incubator set at 300 rpm. Microplate reader-based growth assays were performed using the Tecan Spark multimode microplate reader with 200 ul culture per well. For each experiment, five biological replicates (each with three technical replicates) were used. The concentration of succinate used is calculated according to the carbon equivalence of 4g/L glucose. For growth profiles on succinate three biological replicates (each with three technical replicates) were used. Error bars indicate the standard error of the mean.

Bioinformatics-based prediction of the impact of mutations

The sequence for wild-type succinate dehydrogenase of *E. coli* K-12 was retrieved from the UniProt database under the identifier number P0AC41. The protein structure with PDB ID 1NEK was used for analyzing the impact of mutations. PyMol was used for introducing mutated residues and creating mutant SdhA protein structures.

Succinate dehydrogenase enzyme activity

The assay has been adapted from Spencer ME, 1973 (3). The assay was performed using 75 µg of total protein isolated from log phase cultures. The DCIP reduction with and without

substrate has been used to calculate ΔOD600. The drop in OD600 for every reaction plateaued in 30 minutes.

DNA sequencing and RNA sequencing

DNA sequencing and RNA sequencing were performed using a clone. Total DNA was isolated from an overnight-grown culture, and total RNA was isolated from a mid-log phase culture. Nucleic acid isolation, library preparation, and subsequent analysis were performed as previously described (4).

Metabolic flux mapping was performed as described previously (5).

UPLC analysis for adenylate pool estimation

AXP were extracted from 20 mL mid-log phase culture using the perchloric acid (PCA)-based extraction method as described by Astrid *et al.* (1971) (6). The UPLC analysis was performed using the method adapted from Akhova and Tkachenko (2019) (7). Chromatographic analysis was performed using the Shimadzu-Nexera UPLC system fitted with C18-reverse phase column (Shim-pack C18, 3 mm x 150 mm, 1.9 um). The mobile phase consisted of 50mM potassium phosphate monobasic aqueous salt solution containing 25mM tetrabutylammonium hydrogen sulphate (pH 2.8) with 0.5% acetonitrile. The injection volume was 20 uL, with a flow rate of 0.8 mL/min and UV-absorption was measured at 254 nm.

Quantification and statistical analysis

Prism (GraphPad) version 9 was used for the quantification and generation of plots.

Supplementary References

1. Thomason LC, Costantino N, Court DL. 2007. E. coli genome manipulation by P1 transduction. Curr Protoc Mol Biol Chapter 1:1.17.1–1.17.8.

2. Baba T, Ara T, Hasegawa M, Takai Y, Okumura Y, Baba M, Datsenko KA, Tomita M, Wanner BL, Mori H. 2006. Construction of Escherichia coli K-12 in-frame, single-gene knockout mutants: the Keio collection. Mol Syst Biol 2:2006.0008.

3. Spencer ME, Guest JR. 1973. Isolation and properties of fumarate reductase mutants of Escherichia coli. J Bacteriol 114:563–570.

4. Anand A, Olson CA, Yang L, Sastry AV, Catoiu E, Choudhary KS, Phaneuf PV, Sandberg TE, Xu S, Hefner Y, Szubin R, Feist AM, Palsson BO. 2019. Pseudogene repair driven by selection pressure applied in experimental evolution. Nat Microbiol 4:386–389.

5. Anand A, Olson CA, Sastry AV, Patel A, Szubin R, Yang L, Feist AM, Palsson BO. 2021. Restoration of fitness lost due to dysregulation of the pyruvate dehydrogenase complex is triggered by ribosomal binding site modifications. Cell Rep 35:108961.

6. Adenylate Energy Charge in Escherichia coli During Growth and Starvation. http://dx.doi.org/10.1128/jb.108.3.1072-1086.1971. Retrieved 19 August 2023.

7. Akhova AV, Tkachenko AG. 2019. HPLC–UV method for simultaneous determination of adenosine triphosphate and its metabolites in Mycobacterium smegmatis. Acta Chromatographica 31:45–48.
